# Gut Bacteria Involved in Ellagic Acid Metabolism To Yield Human Urolithin Metabotypes Revealed

**DOI:** 10.1021/acs.jafc.2c08889

**Published:** 2023-02-25

**Authors:** Carlos E. Iglesias-Aguirre, Rocío García-Villalba, David Beltrán, María Dolores Frutos-Lisón, Juan C. Espín, Francisco A. Tomás-Barberán, María V. Selma

**Affiliations:** Laboratory of Food & Health, Research Group on Quality, Safety, and Bioactivity of Plant Foods, CEBAS-CSIC, Murcia 30100, Spain

**Keywords:** polyphenols, metabolism, gut microbiota, interindividual variability, urolithin-producing bacteria

## Abstract

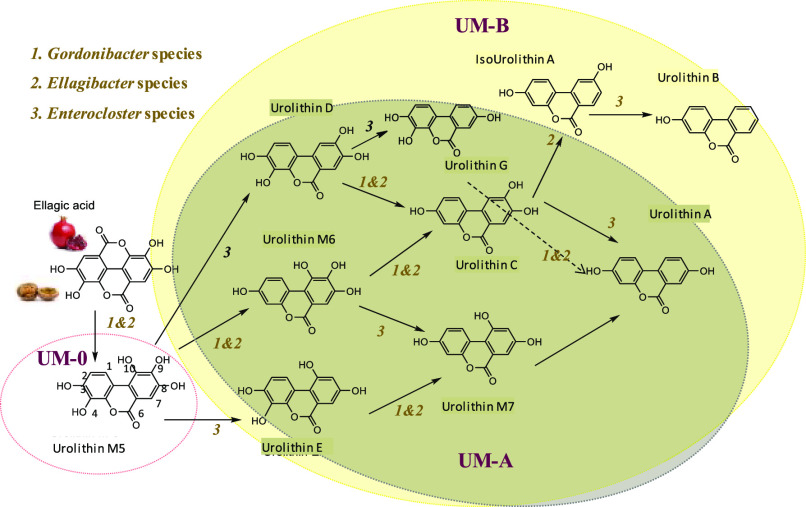

We aimed to elucidate the gut bacteria that characterize the human urolithin metabotypes A and B (UM-A and UM-B). We report here a new bacterium isolated from the feces of a healthy woman, capable of producing the final metabolites urolithins A and B and different intermediates. Besides, we describe two gut bacterial co-cultures that reproduced the urolithin formation pathways upon in vitro fermentation of both UM-A and UM-B. This is the first time that the capacity of pure strains to metabolize ellagic acid cooperatively to yield urolithin profiles associated with UM-A and UM-B has been demonstrated. The urolithin-producing bacteria described herein could have potential as novel probiotics and in the industrial manufacture of bioactive urolithins to develop new ingredients, beverages, nutraceuticals, pharmaceuticals, and (or) functional foods. This is especially relevant in UM-0 individuals since they cannot produce bioactive urolithins.

## Introduction

Urolithins (Uros) have gained recognition as one of the main drivers for the health effects related to the intake of ellagitannins (ETs) and ellagic acid (EA)-rich foods such as nuts, pomegranates, many tropical fruits, and berries. The human gut microbiota converts these polyphenols into Uros. To date, 13 Uros and their conjugated metabolites (glucuronides and sulfates) have been described in different human fluids and tissues (blood, urine, feces, breastmilk, prostate, colon, and breast tissues).^1−3^ Uro production capacity and, consequently, at least partly, the health effects associated with ET consumption vary among individuals because not everyone has the gut bacteria needed to produce all the Uros.^4,5^ Three Uro metabotypes (UMs, i.e., UM-A, UM-B, and UM-0) associated with three different Uro production profiles have been described in western and eastern populations.^6−8^ The Uro production pathways for each UM have been elucidated using human samples and fecal fermentation studies in batch or using a dynamic gastrointestinal simulation model (TWIN-SHIME). Differences in the Uro profiles have been observed between UMs and along the large intestine, showing predominant Uro production in the distal colon region.^9−12^ One of the main differences between the metabolic profiles associated with UMs is the final Uros produced. UM-A individuals only yield Uro-A as the final metabolite of the EA metabolic pathway, whereas UM-B subjects produce Uro-A and, distinctively, IsoUro-A and Uro-B. Finally, UM-0 individuals cannot produce Uros (only the precursor Uro-M5 has been detected so far). Remarkably, the percentage of UM-0 in Spanish and Chinese healthy populations rounds to 10%.^7,8^ UM-0 prevalence could be even higher (60%) in the US population, according to a study with 100 participants, where 33% did not produce or 27% were low producers of Uro-A.^13^ The type of UM depends on the gut microbiota composition of each person.^5,14^ There has been a substantial advance in the research on the specific bacteria involved in Uro production and the compositional and functional characterization of the gut microbiota associated with UMs (UM-A, UM-B, and UM-0).^14−16^ Recent studies demonstrated that more than 30% of the discriminating genera between UM-A and UM-B belonged to the Eggerthellaceae family.^14^ Certainly, genera from this family, such as *Gordonibacter* and *Ellagibacter*, harbor intestinal species that can transform EA into some intermediary Uros.^17−20^ However, the human gut bacteria producing Uro-A and Uro-B (i.e., the main metabolite markers of UM-A and UM-B, respectively), and many intermediate Uros within each UM, are still unknown. Therefore, the present study is aimed to elucidate the gut bacteria that characterize the human UMs.

## Materials and Methods

### Chemicals

As described elsewhere, Uros were chemically synthesized (Villapharma, Murcia, Spain)^10^ or purchased from Dalton Pharma Services (Toronto, Canada). Purity was higher than 95% in all tested compounds.

### Isolation of Uro-Producing Bacteria

A healthy female donor (aged 30), who was previously demonstrated to produce Uros in vivo, provided the stool samples. The study conformed to ethical guidelines outlined in the Declaration of Helsinki and its amendments. The protocol (included in the project AGL2015-64124-R) was approved by the Spanish National Research Council’s Bioethics Committee (Spain). The donor gave written informed consent following the Declaration of Helsinki. As explained elsewhere, Uros were identified in feces and urine after walnut consumption.^21^ The feces were prepared to isolate Uro-producing bacteria following a protocol previously described with some modifications.^19,20^ Briefly, after 1/10 (w/v) fecal dilution in nutrient broth (Oxoid, Basingstoke, Hampshire, UK) supplemented with 0.05% l-cysteine hydrochloride (PanReac Química, Barcelona, Spain), the filtrated sample was homogenized and further diluted in Wilkins–Chalgren anaerobe medium (WAM, Oxoid). The metabolic activity was evaluated by adding to the broth Uro-C (Dalton Pharma Services) dissolved in propylene glycol (PanReac Química SLU, Barcelona, Spain) to reach a final concentration of 15 μM. After anaerobic incubation, a portion of the culture, having metabolic activity, was seeded on WAM agar. Colonies were collected and inoculated into 5 mL of WAM containing 15 μM Uro-C, and after incubation, their capacity to convert Uro-C was assayed. Uro-C-transforming colonies were subcultured until single strains were isolated. The isolation procedure and plate incubation were achieved in an anaerobic chamber (Concept 400, Baker Ruskin Technologies Ltd., Bridgend, South Wales, UK) at 37 °C. Samples (5 mL) were prepared for HPLC–DAD–MS analyses of Uros. We isolated pure bacterial cultures (*Enterocloster bolteae* strain CEBAS S4A9), which showed the capacity to transform Uro-C. This strain was phylogenetically identified, and its metabolic characteristics were analyzed as described below.

### Identification of the Isolated Uro-Producing Bacteria

The almost-complete 16S rRNA gene sequence of the isolated bacterial strain (*E. bolteae* CEBAS S4A9) and the phylogenetic analysis were achieved as previously described.^20^ A phylogenetic tree, including the isolated strain *E. bolteae* CEBAS S4A9, the most closely related species and known Uro-producing genera (*Gordonibacter* and *Ellagibacter*), was constructed using the neighbor-joining treeing method.^19^

### Conversion Testing of EA and Intermediary Uros

The isolated strain *E. bolteae* CEBAS S4A9 and representative strains of the closest relatives (*E. bolteae* DSM 29485, DSM 15670^T^, *Enterocloster asparagiformis* DSM 15981^T^, *Enterocloster citroniae* DSM 19261^T^, and *Enterocloster clostridioformis* DSM 933^T^) obtained from the DSMZ culture collection were used to investigate their capacity to produce final Uros in the presence of EA and other Uro intermediaries. Briefly, isolated and DSMZ strains were separately incubated on a WAM agar plate for 6 days. A single colony was cultivated in a 5 mL WAM tube. Diluted inoculum (2 mL) was transferred to WAM (20 mL), obtaining an initial load of 10^4^ CFU mL^–1^. EA, Uro-M6, Uro-D, Uro-C, Uro-A, IsoUro-A, and Uro-B were dissolved in propylene glycol and added to the 20 mL cultures to obtain a final concentration of 15 μM each. After incubation in an anoxic environment at 37 °C, aliquots (5 mL) were taken periodically for high-performance liquid chromatography (HPLC) analyses as described below.

### In Vitro Conversion of EA with Gut Bacteria To Reproduce UMs

*Gordonibacter urolithinfaciens* DSM 27213^T^, *Ellagibacter isourolithinifaciens* DSM 104140^T^ obtained from the DSMZ culture collection, and the isolated strain *E. bolteae* CEBAS S4A9 were cultivated anaerobically in 5 mL WAM tubes. First, 2 mL of a diluted aliquot of *G. urolithinfaciens* DSM 27213^T^ and *E. bolteae* CEBAS S4A9 strains was transferred to WAM (100 mL). Similarly, 2 mL of diluted aliquots of *E. isourolithinifaciens* DSM 104140^T^ and *E. bolteae* CEBAS S4A9 strains was transferred to WAM (100 mL). Finally, EA dissolved in propylene glycol was added to the 100 mL cultures to obtain a final concentration of 25 μM. During incubation in an anoxic environment at 37 °C, aliquots (5 mL) were taken for HPLC analyses as described below. Incubations were made in triplicate, and the experiment was repeated twice.

### Sample Clean-Up and HPLC–DAD–MS Analyses

As previously described, aliquots (5 mL) collected during the incubation of single and combined bacterial strains were extracted and analyzed by HPLC–DAD–ESI-Q (MS).^19^ Briefly, fermented medium (5 mL) was extracted with ethyl acetate (5 mL) (Labscan, Dublin, Ireland), acidified with 1.5% formic acid (PanReac), vortexed for 2 min, and centrifuged at 3500*g* for 10 min. The organic phase was separated and evaporated, and the dry samples were then re-dissolved in methanol (250 μL) (Romil, Barcelona, Spain). An HPLC system (1200 Series, Agilent Technologies, Madrid, Spain) equipped with a photodiode-array detector (DAD) and a single quadrupole mass spectrometer detector in series (6120 Quadrupole, Agilent Technologies, Madrid, Spain) was used. Calibration curves were obtained for EA, Uro-M6, Uro-D, Uro-C, Uro-A, Uro-B, and IsoUro-A with good linearity (*R*^2^ > 0.998).

## Results

### Identification of Uro-Producing Bacteria

**Figure 1:**
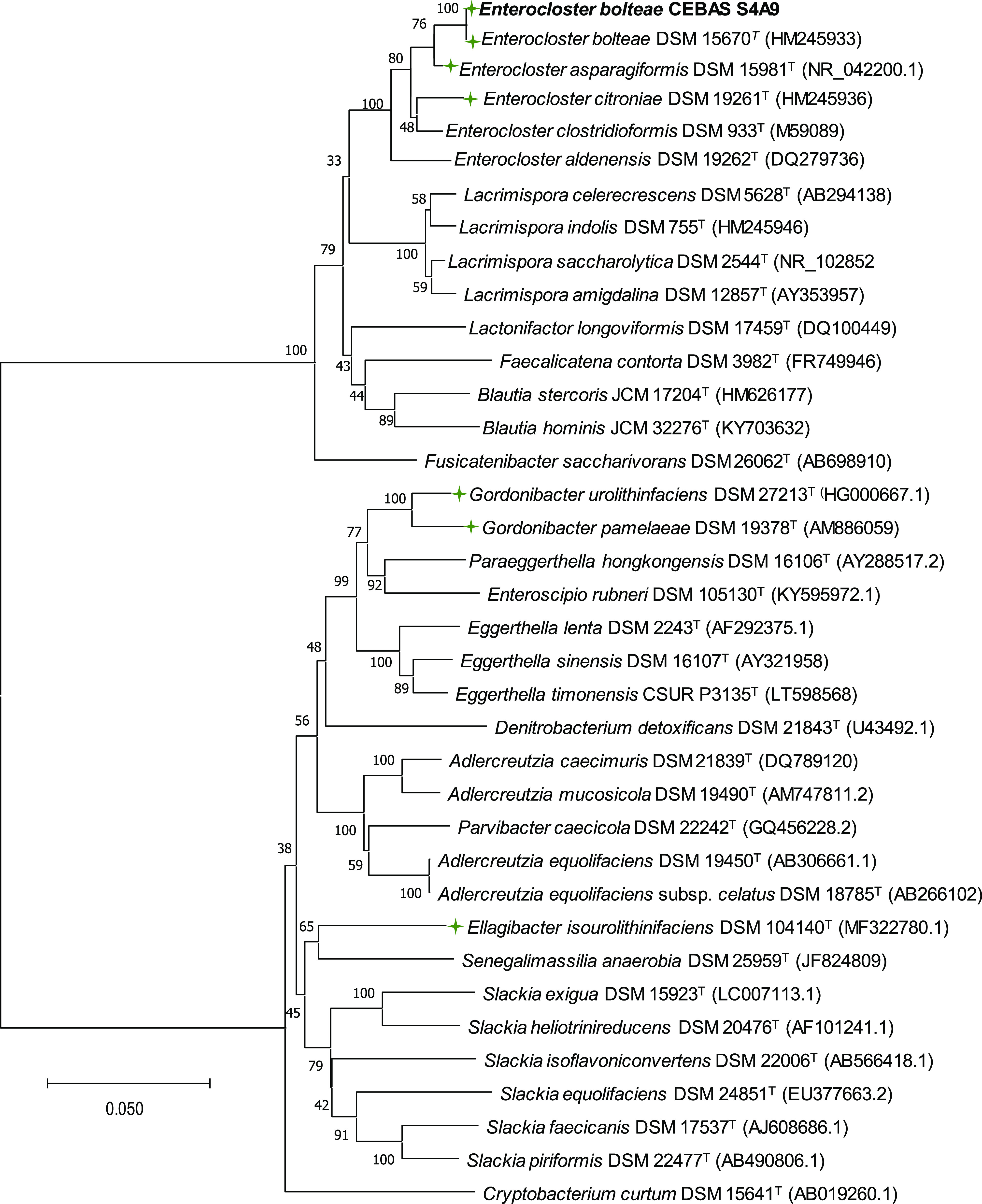
Phylogenetic tree showing the relationship between the strain *E. bolteae* CEBAS S4A9 and other Uro-producing bacteria (green color). The tree was constructed using the neighbor-joining method based on 16S rRNA gene sequences. The distance matrix was calculated by the Jukes–Cantor method. GenBank accession numbers are presented in parentheses. Bar, 0.05 substitutions per nucleotide position. Numbers at nodes (≥70%) indicate support for internal branches within the tree obtained by bootstrap analysis (percentages of 500 re-samplings).

One bacterial strain isolated from a human fecal sample, named strain CEBAS S4A9, obtained from a 1:10^4^ dilution plated on WAM agar, showed the capacity to convert Uro-C into Uro-A under anaerobic conditions. A nearly complete 16S rRNA gene sequence (1389 bp) was obtained for isolating CEBAS S4A9. The sequence was aligned with the closest accepted members of this family. The phylogenetic tree, representing minimum evolutionary distances (Jukes–Cantor), showed that strain CEBAS S4A9 grouped with the other members of the *Enterocloster* genus (Figure 1). The closest relatives of strain CEBAS S4A9 are *E. bolteae* DSM 15670^T^ (99.8% 16S rRNA gene sequence similarity), *E. asparagiformis* DSM 15981^T^ (98.0%), *E. citroniae* DSM 19261^T^ (97.0%), and *E. clostridioformis* DSM 933^T^ (97.7%). A higher distance was observed with other known Uro-producing bacteria in the phylogenetic tree, i.e., *G. pamelaeae* DSM 19378^T^ (80.9%), *Gordonibacter urolithinfaciens* DSM 27213^T^ (78.2%), and *Ellagibacter isourolithinifaciens* DSM 104140^T^ (80.0%).

### Analysis of Uros Produced by *Enterocloster* Species

**Figure 2:**
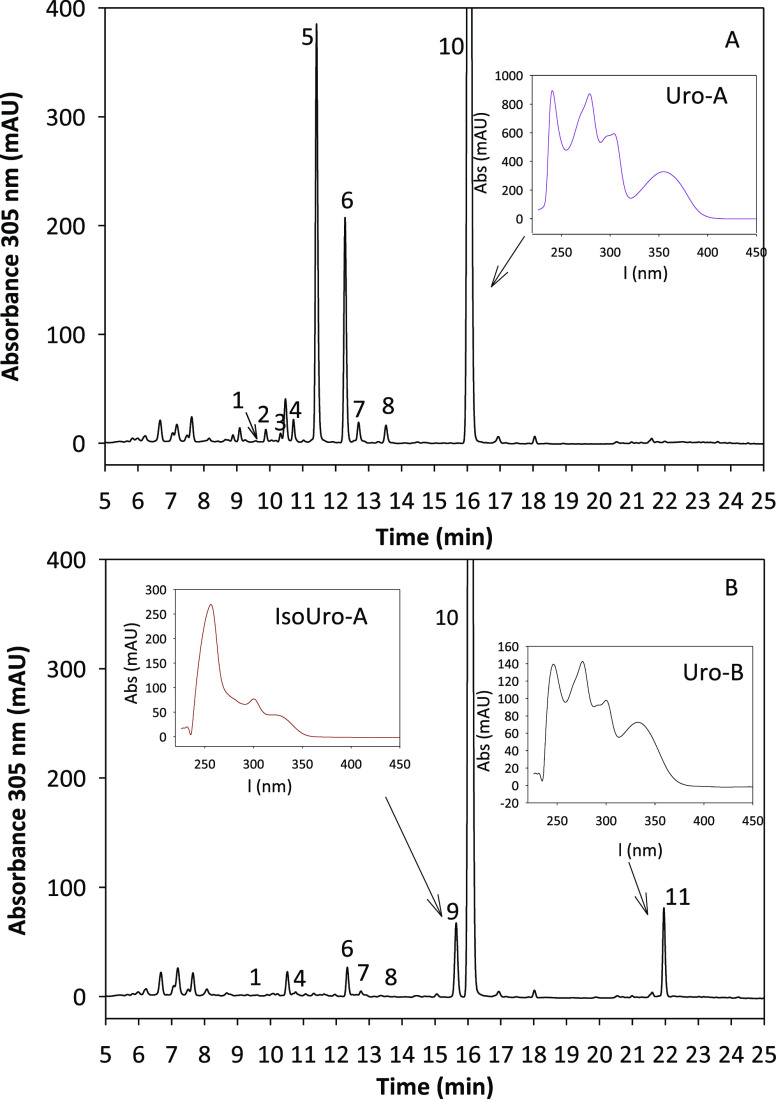
HPLC–DAD chromatogram of in vitro metabolism of EA by (A) *G. urolithinfaciens* DSM 27213^T^ and *E. bolteae* CEBAS S4A9 strains, which mimic the Uro metabotype A (UM-A) and by (B) *E. isourolithinifaciens* DSM 104140^T^ and *E. bolteae* CEBAS S4A9 strain co-culture, which mimics the Uro metabotype B (UM-B). 1: Uro-M5; 2: Uro-D; 3: Uro-E; 4: EA; 5: Uro-M6; 6: Uro-C; 7: Uro-G; 8: Uro-M7; 9: IsoUro-A; 10: Uro-A; 11: Uro-B.

The HPLC–MS analyses showed that, in contrast to *G. urolithinfaciens* DSM 27213^T^ and *E. isourolithinifaciens* DSM 104140^T^, the isolate *E. bolteae* CEBAS S4A9 and the closest relatives (*E. bolteae* DSM 29485, DSM 15670^T^, *E. asparagiformis* DSM 15981^T^, *E. citroniae* DSM 19261^T^, and *E. clostridioformis* DSM 933^T^) did not metabolize EA (Table 1). However, all *Enterocloster* species tested, except *E. clostridioformis* DSM 933^T^, metabolized Uro-M6 to other Uros, such as Uro-A, via Uro-M7. Table 1 shows the specific Uros produced by each microbial species after incubation with the different precursors. *G. urolithinfaciens* DSM 27213^T^ and *E. isourolithinifaciens* DSM 104140^T^ also transformed Uro-M6, but *G. urolithinfaciens* rendered Uro-C, whereas *E. isourolithinifaciens* produced IsoUro-A via Uro-C. Uro-D was also transformed by most of the *Enterocloster* species tested, rendering a novel metabolite that we named urolithin G (Uro-G; 3,4,8-trihydroxy-urolithin), whose structure was recently established.^22^ Uro-G showed an Rt at 12.58 min that did not coincide, under the same assay conditions, with the already known trihydroxy-urolithins, Uro-C (Rt 12.44 min), Uro-CR (Rt 13.17 min), and Uro-M7 (Rt 13.59 min) (Figure 2), suggesting a new metabolite (7 in Figure 2A,B). In contrast, *G. urolithinfaciens* DSM 27213^T^ transformed Uro-D until Uro-C, whereas *E. isourolithinifaciens* DSM 104140^T^ transformed Uro-D until IsoUro-A via Uro-C (Table 1). Most of the *Enterocloster* species tested completely transformed Uro-C into Uro-A except *E. clostridioformis*, which gave negative reactions for Uro production. *E. isourolithinifaciens* DSM 104140^T^ also transformed Uro-C but only until IsoUro-A. Unlike *E. isourolithinifaciens* DSM 104140^T^ and *G. urolithinfaciens* DSM 27213^T^, *Enterocloster* species further converted IsoUro-A into Uro-B. Interestingly, the type strain of *E. bolteae* did not transform IsoUro-A into Uro-B, unlike the other strains of *E. bolteae* tested (CEBAS S4A9 and DSM 29485) (Table 1). None of these bacterial strains dehydroxylated Uro-A or Uro-B (data not shown). As reported previously, all metabolites were identified by direct comparison (UV spectra and MS) with standards and confirmed by their spectral properties and molecular masses.^10^

**Table 1:** Main Metabolites Produced after Incubating Bacterial Strains with EA and Uros

	EA	Uro-M6	Uro-D	Uro-C	IsoUro-A	Uro-A
*E. bolteae* CEBAS S4A9		Uro-M7	Uro-G	Uro-A	Uro-B	
*E. bolteae* DSM 15670^T^		Uro-M7		Uro-A		
*E. bolteae* DSM 29485		Uro-M7	Uro-G	Uro-A	Uro-B	
*E. asparagiformis* DSM 15981^T^		Uro-A	Uro-G	Uro-A	Uro-B	
*E. citroniae* DSM 19261^T^		Uro-A	Uro-G	Uro-A	Uro-B	
*E. clostridioformis* DSM 933^T^						
*G. urolithinfaciens* DSM 27213^T^	Uro-M5, Uro-M6, Uro-C	Uro-C	Uro-C			
*E. isourolithinifaciens* DSM 104140^T^	Uro-M5, Uro-M6, Uro-C, IsoUro-A	Uro-C, IsoUro-A	Uro-C, IsoUro-A	IsoUro-A		

### In Vitro Catabolism of EA by Human Gut Bacteria Co-Culture Reproducing UMs

The in vitro co-culture of *G. urolithinfaciens* DSM 27213^T^ and *E. bolteae* CEBAS S4A9 strains (co-culture 1) and that of *E. isourolithinifaciens* DSM 104140^T^ and *E. bolteae* CEBAS S4A9 strains (co-culture 2) were followed to study their Uro production patterns from EA (Figure 2). The HPLC–DAD chromatogram at 15 h of incubation showed the production of Uro-M5, Uro-D, Uro-E, Uro-M6, Uro-C, Uro-G, Uro-M7, and Uro-A from EA by the bacterial co-culture 1 (potential UM-A reproducer) (Figure 2A). In the case of the bacterial co-culture 2 (potential UM-B reproducer), the HPLC–DAD chromatogram showed the production of Uro-M5, Uro-C, Uro-G, Uro-M7, IsoUro-A, Uro-A, and Uro-B from EA (Figure 2B). In both chromatograms, Uro-M5 was barely detected. Uro-E and the novel Uro-G were quantified using the Uro-M7 standard, whereas Uro-M5 was quantified using Uro-M6 as there were no standards for these Uros.

**Figure 3:**
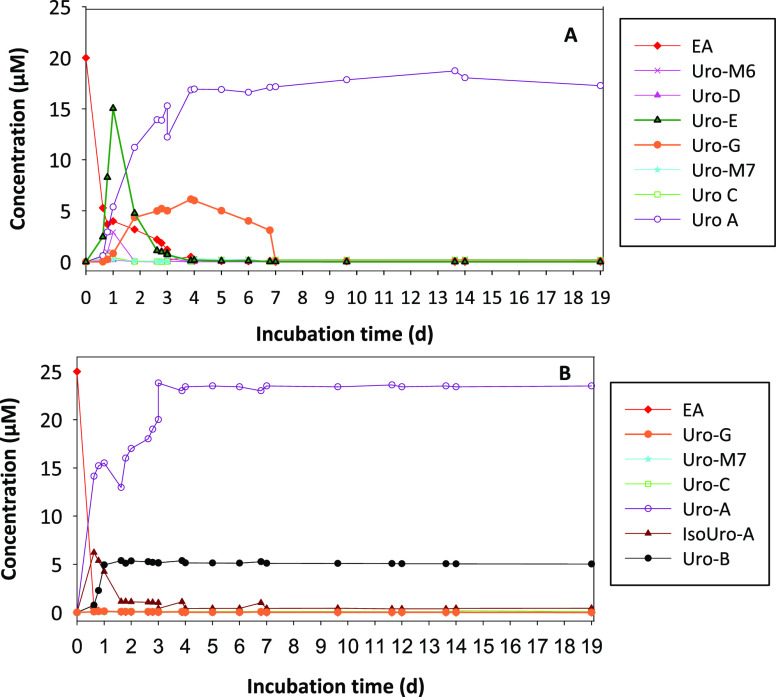
Time course production of Uros from EA. (A) Metabolism of EA by *G. urolithinfaciens* DSM 27213^T^ and *E. bolteae* CEBAS S4A9 strain co-culture. (B) Metabolism of EA by *E. isourolithinifaciens* DSM 104140^T^ and *E. bolteae* CEBAS S4A9 strain co-culture.

When EA was incubated with co-culture 1 (potential UM-A reproducer), Uros started to be detected after 15 h of incubation (Figure 3A). Uro-D was only detected at this time. Uro-M6 and Uro-E (tetrahydroxy-urolithins) also appeared at 15 h with a concentration of 3.34 and 0.21 μM, respectively (Figure 3A). Regarding trihydroxy-urolithins, Uro-C also peaked at 15 h of incubation and reached a plateau. Uro-M7 and Uro-G started to be detected at 15 h of incubation and then progressively decreased. Concerning dihydroxy-urolithins, only Uro-A was detected, reaching a maximum concentration of 18.71 μM (Figure 3A). Most EA disappeared on the third day of incubation, with remaining nonmetabolized EA concentrations in the medium being lower than 0.06 μM after 5 days (Figure 3A). When EA was incubated with co-culture 2 (potential UM-B reproducer), EA started to be converted to Uro-M7 and Uro-C via Uro-M5 and Uro-M6. The maximal Uro-M7, Uro-C, and Uro-G concentrations were achieved on the third day. Then, a plateau was maintained but only in the case of Uro-C. Uro-A started to be detected at 15 h of incubation and reached a concentration of 18.86 μM (Figure 3B). Similarly, IsoUro-A and Uro-B started to be detected at 15 h of incubation, and then a plateau was reached. Most EA was metabolized, and no EA was detected after 7 days (Figure 3B).

## Discussion

**Figure 4:**
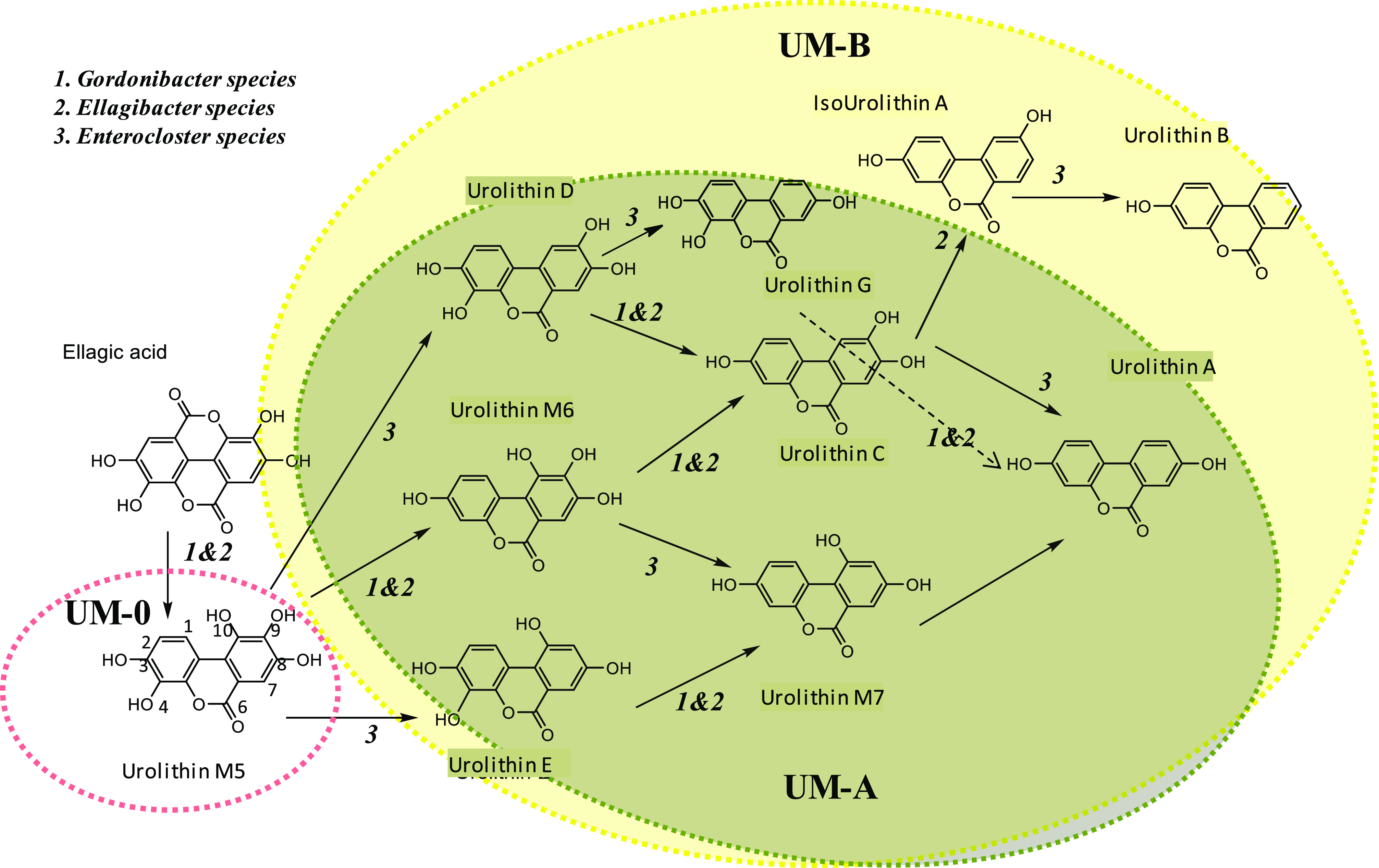
Proposed metabolic pathway of EA by strains from *Gordonibacter* (1), *Ellagibacter* (2), and *Enterocloster* (3) genera including the isolate *E. bolteae* CEBAS S4A9. (1) *Gordonibacter urolithinfaciens* and *G. pamelaeae*; (2) *Ellagibacter isourolithinifaciens*; (3) *Enterocloster bolteae*, *E. asparagiformis*, and *E. citroniae*.

The specific gut microbial ecology of UMs can indirectly affect the health benefits attributed to ETs and EA consumption.^14^ In the present study, we have revisited the metabolic capacity of known Uro-producing genera (*Gordonibacter* and *Ellagibacter*) using different intermediary Uros as substrates. The genus *Gordonibacter*, predominant in UM-A individuals,^14^ metabolizes EA into Uro-M5, Uro-M6, and Uro-C.^17,18^*Ellagibacter*, another genus from the Eggerthellaceae family, predominant in UM-B individuals, can also convert EA into some Uros (Uro-M5, Uro-M6, Uro-C, and IsoUro-A).^19,20^ In the present study, we observed that the *Gordonibacter* and *Ellagibacter* genera also converted Uro-D and Uro-M6 into Uro-C because of their 4- and 10-dehydroxylase activities, respectively (Table 1 and Figure 4). However, the *Gordonibacter* and *Ellagibacter* strains could not produce Uro-A from Uro-C, neither Uro-B from Uro-A nor any other Uro conversion involving the dehydroxylation activity at the 9-position, including the conversion of Uro-M6 into Uro-M7, or that of Uro-D into the novel Uro-G, which is described here for the first time (Figure 4). Similarly, *Gordonibacter* and *Ellagibacter* did not produce the intermediaries Uro-E or Uro-M7 from EA because of their lack of dehydroxylation activity at the 9-position. Consequently, other unknown bacteria from the gut were necessary to complete the EA metabolism associated with human UMs (Table 1 and Figure 4).

We report here a new bacterium isolated from the feces of a healthy woman, capable of producing the final metabolites Uro-A and Uro-B from Uro-C and IsoUro-A, respectively. The comparison of the 16S rRNA gene sequence of the strain showed that the isolate belongs to the *Enterocloster bolteae* species (99.8% similarity with the type strain *E. bolteae* DSM 15670) from the family Lachnospiraceae. Before creating the family Lachnospiraceae, this large group was recognized as the *Clostridium* cluster XIVa or *Clostridium coccoides* group. The clade with *C. bolteae*, *Clostridium asparagiformis*, *Clostridium citroniae*, *Clostridium clostridioforme*, and *Clostridium aldenensis* has recently been reclassified as *Enterocloster* gen. nov., and the species as *Enterocloster bolteae* comb. nov., *Enterocloster asparagiformis* comb. nov., *Enterocloster citroniae* comb. nov., *Enterocloster clostridioformis* comb. nov., and *Enterocloster aldensis* comb. nov., respectively.^23^ Some Lachnospiraceae species, such as *Butyrivibrio* and *Blautia*, are known for being benign members of gut microbiomes and their plant-degrading capabilities, including the metabolism of phenolic compounds.^5^

We show here that the isolate *E. bolteae* CEBAS S4A9 and its closest relatives, such as *E. bolteae* DSM 29485, DSM 15670^T^, *E. asparagiformis* DSM 15981^T^, and *E. citroniae* DSM 19261^T^, produced the final Uros Uro-A and Uro-B. (Table 1). However, none could metabolize EA, not even into intermediate Uros such as Uro-M5, unlike *Gordonibacter* and *Ellagibacter*. Therefore, although phylogenetically far, genera from these two families (Lachnospiraceae and Eggerthellaceae) have complementary activities in the EA catabolism to produce Uros. *Gordonibacter* transformed EA into Uros by lactone-ring cleavage, decarboxylation, and further catechol dehydroxylations at 4- and 10-positions. *Ellagibacter* shared with *Gordonibacter* the lactone-ring cleavage and decarboxylation but dehydroxylated at the 4-, 8-, and 10-positions (Table 1 and Figure 4). *Ellagibacter* did not produce Uro-B from Uro-G or Uro-A despite having 8-dehydroxylase capacity. In contrast, it can produce Uro-A from Uro-G. This suggests that it can only dehydroxylate on catechol rings. On the contrary, the *Enterocloster* genera catalyzed the dehydroxylation of hydroxyl groups at 9- and 10-positions, regardless of whether they were in a catechol ring (Table 1 and Figure 4). Uro-G was only obtained after Uro-D incubation with the *Enterocloster* species that harbor 9-dehydroxylase activity. This supports that Uro-G corresponds to 3,4,8-trihydroxy-urolithin.^22^

We tested and patented two bacterial combinations to reproduce the Uro profiles that characterize the human UM-A and UM-B, i.e., group 1 combined *G. urolithinfaciens* DSM 27213^T^ and *E. bolteae* CEBAS S4A9 strains, whereas group 2 combined *E. isourolithinifaciens* DSM 104140^T^ and *E. bolteae* CEBAS S4A9 strains.^22^ The metabolic capabilities of the two co-cultures were followed in vitro to study the time course production of the potential intermediate catabolites in the route from EA to Uro-A or Uro-B (Figure 3). Besides, the similarities with the Uro profiles of UM-A and UM-B individuals were also analyzed. Uro metabolic profiles of UM-A individuals described in vivo^6−8^ and in fecal fermentation studies^9−12^ showed a lack of 8-dehydroxylase activity and were consistent with those found in vitro during the incubation of EA with co-culture 1 (Figures 2A and 3A). In the case of co-culture 2 (Figures 2B and 3B), the Uro profile obtained resembled the metabolic profile of UM-B individuals described in vivo^6−8^ and in human fecal fermentation studies.^9−12^ In the present study, bacterial combinations 1 and 2 included the *E. bolteae* CEBAS S4A9 strain. However, similar results would have been obtained with other *Enterocloster* species such as *E. bolteae* (strain CEBAS S4A9, DSM 15670^T^), *E. asparagiformis* DSM 15981^T^, and *E. citroniae* DSM 19261^T^ because of their implication in Uro metabolism, unlike *E. clostridioformis* DSM 933^T^ (Table 1).

We report here for the first time the capacity of pure strains to metabolize EA cooperatively to render Uro profiles associated with UM-A and UM-B. The Uro-producing bacteria described herein could have potential as novel probiotics and in the industrial manufacture of bioactive Uros to develop new ingredients, beverages, nutraceuticals, pharmaceuticals, and (or) functional foods. This is especially relevant in those individuals with UM-0 since they cannot produce bioactive Uros. Uro-A administration has recently been assayed for safety requirements and Generally Recognized as Safe (GRAS) by the Food and Drug Administration (FDA: 20-12-2018. GRAS Notice No. GRN 000791).^24^ The impact and safety of oral supplementation with Uro-A were recently investigated in a randomized clinical trial in middle-aged adults. Results showed that oral administration of Uro-A improved muscle strength and exercise performance measures accompanied by an impact on mitochondrial biomarkers.^13^ However, in the case of Uro-producing bacteria, further research is necessary to probe well-established health effects on the host as well as safety requirements before being considered among the next-generation probiotics.
